# Gut microbiota-mediated protection against influenza virus subtype H9N2 in chickens is associated with modulation of the innate responses

**DOI:** 10.1038/s41598-018-31613-0

**Published:** 2018-09-04

**Authors:** Alexander Yitbarek, Khaled Taha-Abdelaziz, Douglas C. Hodgins, Leah Read, Éva Nagy, J. Scott Weese, Jeff L. Caswell, John Parkinson, Shayan Sharif

**Affiliations:** 1 Department of Pathobiology, Ontario Veterinary College, University of Guelph, Ontario, N1G 2W Canada; 2 Pathology Department, Faculty of Veterinary Medicine, Beni-Suef University, Al Shamlah, 62511 Beni-Suef Egypt; 3 Department of Computer Science, University of Toronto, Toronto, M5S 3G4 Canada; 4 Division of Molecular Structure and Function, Research Institute, Hospital for Sick Children, Toronto, Ontario M5G 1X8 Canada; 5 Departments of Biochemistry and Molecular Genetics, University of Toronto, Toronto, M5S 1A8 Canada

## Abstract

Commensal gut microbiota plays an important role in health and disease. The current study was designed to assess the role of gut microbiota of chickens in the initiation of antiviral responses against avian influenza virus. Day-old layer chickens received a cocktail of antibiotics for 12 (ABX-D12) or 16 (ABX-D16) days to deplete their gut microbiota, followed by treatment of chickens from ABX-12 with five *Lactobacillus* species combination (PROB), fecal microbial transplant suspension (FMT) or sham treatment daily for four days. At day 17 of age, chickens were challenged with H9N2 virus. Cloacal virus shedding, and interferon (IFN)-α, IFN-β and interleukin (IL)-22 expression in the trachea, lung, ileum and cecal tonsils was assessed. Higher virus shedding, and compromised type I IFNs and IL-22 expression was observed in ABX-D16 chickens compared to control, while PROB and FMT showed reduced virus shedding and restored IL-22 expression to levels comparable with undepleted chickens. In conclusion, commensal gut microbiota of chickens can modulate innate responses to influenza virus subtype H9N2 infection in chickens, and modulating the composition of the microbiome using probiotics- and/or FMT-based interventions might serve to promote a healthy community that confers protection against influenza virus infection in chickens.

## Introduction

Avian influenza viruses (AIV) are categorized into high (HPAI) and low (LPAI) pathogenicity viruses based on disease severity. Some LPAI such as H9N2 subtype pose a significant public health threat as they can replicate in permissive mammalian tissues without prior adaptation^1–3^. Furthermore, previous reassortant isolates in humans, such as H5N1, H7N9, H10N8 and H5N6, have been shown to carry a partial or a whole set of internal genes from avian H9N2 viruses^4–7^. Therefore, control of avian H9N2 influenza virus in poultry can have a significant positive impact on the poultry industry and also public health.

As a LPAI virus, AIV subtype H9N2 has tropism for several tissues, including tissues of the upper respiratory tract and gastrointestinal tract (GIT) of chickens^8^. Even though the interplay between bacterial pathogens and commensal gut microbiota of chickens and other animals has been studied extensively, there is a paucity of research on the role of commensal gut microbiota in viral infections. We have recently shown that infection of chickens with AIV subtype H9N2 results in changes in the composition of the fecal microbiota without restoration to a pre-infection microbial composition after the virus was undetectable^9^. Marek’s disease virus (MDV) infection of chickens has also been shown to result in a shift in the composition of gut microbiota with involvement of the immune system and metabolic pathways^10,11^. These studies highlight a role for gut microbiota of chickens in viral infections. Understanding the mechanism of immunity initiated by commensal gut microbiota is of paramount importance to utilize commensal gut microbiota in the form of probiotics or other strategies for shifting the composition to a state where it can allow the host to control AIV infection and shedding, thereby reducing transmission.

The GIT microbiota plays an important role in the induction and regulation of host responses to various pathogens including bacteria^12–14^, fungi^15^ and viruses^16–18^. Recently, we showed that changes in the composition of the gut microbiota towards a dysbiotic condition resulted in higher oropharyngeal and cloacal shedding of AIV subtype H9N2 in chickens, which was also associated with compromised type I interferon (IFN) expression^19^. However, altering the composition of gut microbiota using probiotics has shown beneficial effects on immunity to influenza virus infection^20,21^. For instance, oral administration of a human isolate of *Bifidobacterium longum* MM-2 to influenza-infected mice reduced influenza virus associated mortality and inflammatory responses in the lower respiratory tract^20^. The major mechanisms involved were shown to be the activation of natural killer (NK) cells both in the lungs and spleen and increased expression of various cytokines in the lungs^20^. Furthermore, oral administration of heat-killed *Lactobacillus plantarum* L-137 to mice infected with influenza virus enhanced protection against the virus and reduced virus titre via a type I interferon dependent mechanism^21^. Microbes and antigens of microbial origin are sensed by the GIT resident dendritic cells (DCs) resulting in migration of DCs to the draining lymph nodes followed by activation of T cell subsets and production of various cytokines and homing molecules, which are important for the trafficking of T cells to the respiratory system during infection^16,22^. Therefore, understanding the mechanisms of innate immunity against AIV in chickens initiated by the gut microbiota could allow for the development of effective probiotics to enhance immunity against viruses. We hypothesized that dysbiosis of the gut microbiota of chicken results in higher AIV subtype H9N2 shedding, compromised innate responses, and a recovery from dysbiosis using probiotics and fecal microbial transplant (FMT) administration can result in the recovery of innate responses against the virus and reduced virus shedding. Therefore, in this study, a model was used in which a cocktail of antibiotics (ABX) was administered to chickens to induce dysbiosis of the gut microbiota. Subsequently, either a combination of five *Lactobacillus* species (PROB) or FMT was used to reconstitute the gut microbiota and to assess the ability of PROB and FMT to reverse the effects of antibiotics on the chicken immune system.

## Results

### Gut microbial community composition

**Figure 1:**
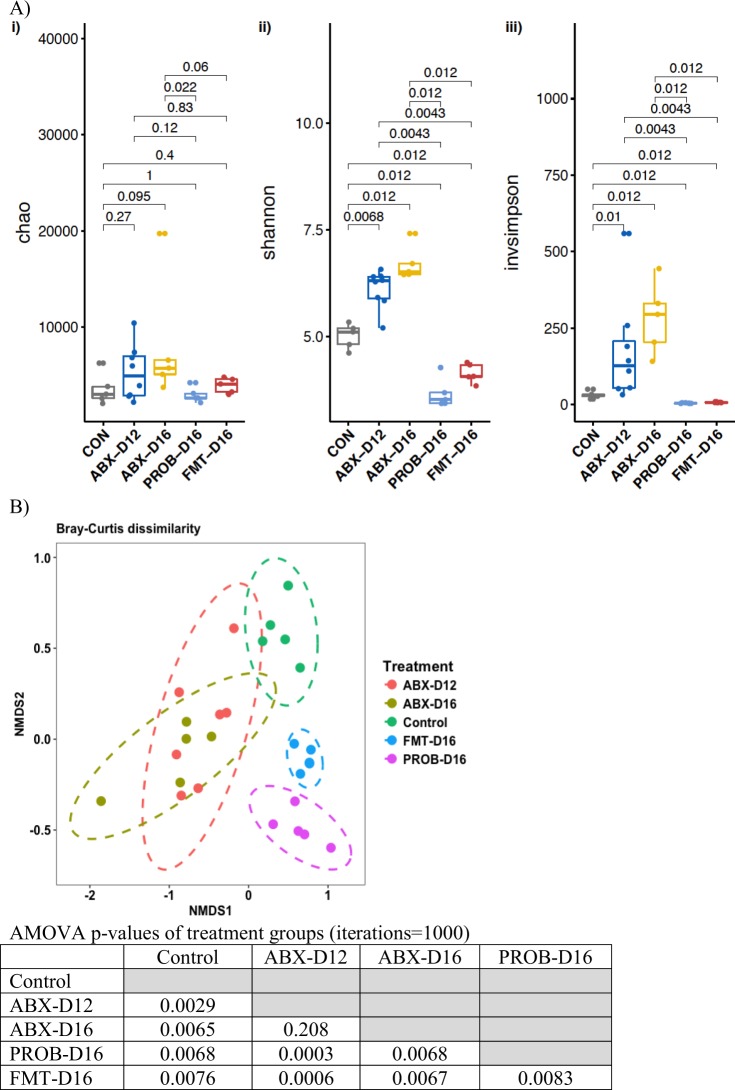
Diversity measures of cecal microbiota of chickens. (**A**) Alpha diversity comparisons of cecal microbiota of chickens. (**B**) Non-metric multi-dimensional scaling (NMDS) plot illustrating the chicken cecal microbiome beta-diversity among treatments, and AMOVA p-values demonstrating significant differences in the centroid of clouds of treatments in the NMDS plot, where significant differences were considered at a P < 0.05.

Alpha diversity measures showed that antibiotics treatment for both 12 and 16 days resulted in higher evenness (Shannon diversity index) and diversity (inverse Simpson’s) compared to control (P < 0.012, ANOVA), while PROB and FMT treatments resulted in significantly lower evenness compared to control, and both antibiotics treatments (P < 0.012, ANOVA) (Fig. 1A). Non-metric multi-dimensional scaling performed with Bray Curtis dissimilarities showed significant difference in the clustering among treatments except for those with only antibiotics administration. Analysis of molecular variance (AMOVA) for measuring beta-diversity showed that the control group was significantly different from all other treatments (P < 0.01, AMOVA). Both ABX groups showed a significant difference in community composition compared to groups treated with probiotics or FMT following antibiotic treatments (P < 0.01, AMOVA). Furthermore, there was a significant difference in beta diversity between chickens treated with probiotics and FMT (after antibiotic treatment, for both groups) (P = 0.0083, AMOVA) (Fig. 1B).

**Figure 2:**
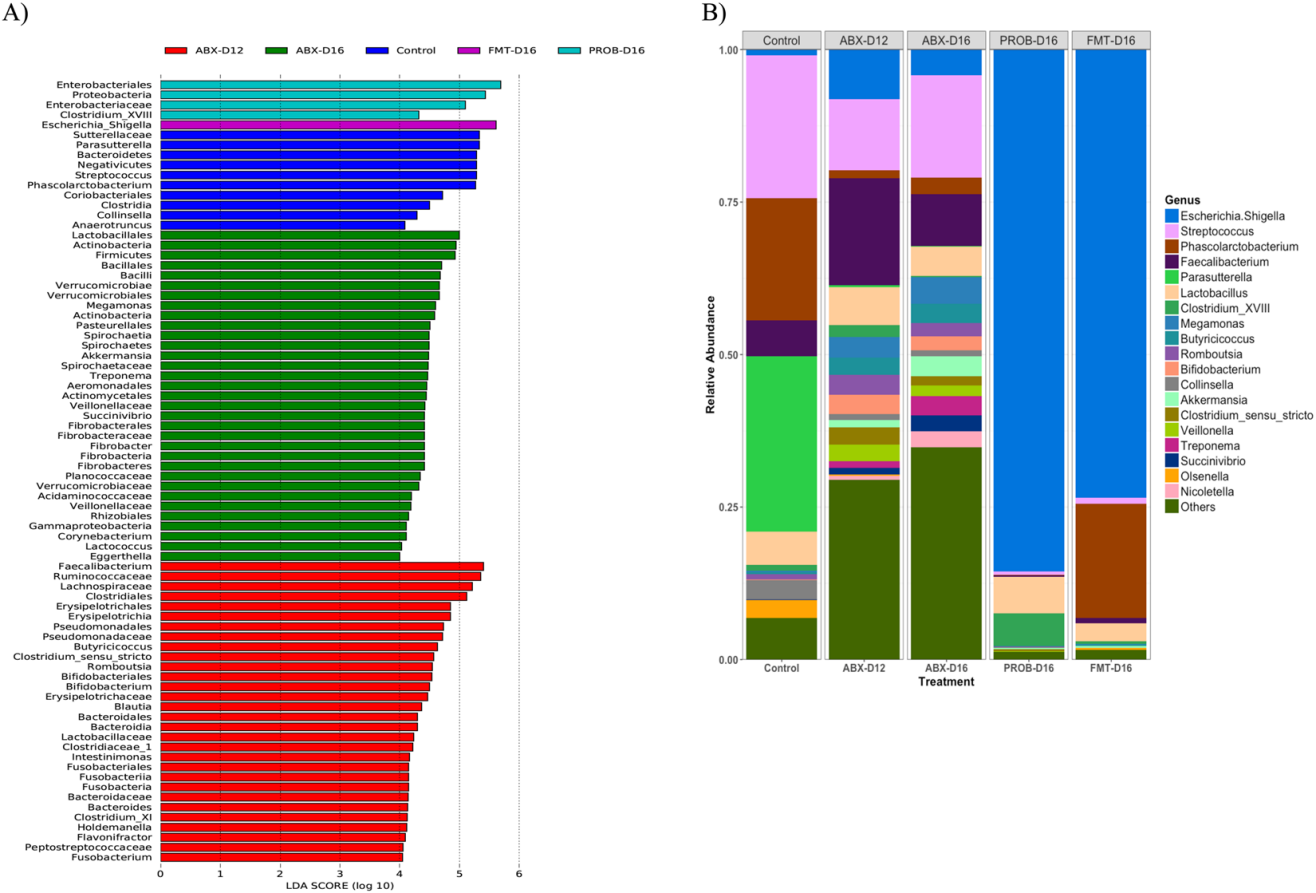
Differential enrichment of cecal microbiota of chickens. (**A**) Linear discriminant analysis (LDA) Effect Size (LefSe) analysis showing those OTUs that were significantly differentially abundant among groups, ranked by effect size (P = 0.05, LDA score of at least 4). (**B**) Stacked bars showing relative abundance at the genus level.

Supervised comparison of control and H9N2-infected chickens using the linear discriminant analysis (LDA) Effect Size (LEfSe) algorithm (P = 0.05, LDA score of at least 4) to identify differential enrichment of microbes showed that the control group was enriched with phylum Bacteroidetes, class Negativicutes and Clostridia, order Coriobacteriales, and genera *Streptococcus*, *Phascolarctobacterium*, *Anaerotruncus*, *Parasutterella* and *Collinsella*. Treatment of chickens with antibiotics for 12 days resulted in enrichment with class Erysipelotrichia, Bacteroidia, order Clostridiales, Erysipelotrichales, Pseudomonadales, Bifidobacteriales and Bacteroidales, family *Ruminococcaceae*, *Lachnospiraceae*, *Erysipelotrichaceae*, *Clostridiaceae* and *Lactobacillaceae*, and genera *Blautia*, *Clostridium* cluster XI, *Faecalibacterium*, *Flavonifractor*, *Romboutsia*, *Butyricicoccus* and *Bifidobacterium*. PROB chickens showed enrichment with phylum Proteobacteria, order Enterobacteriales, family Enterobacteriaceae, and genus *Clostridium* cluster *XVIII*. In both PROB and FMT treatments, an enrichment with the genus *Escherichia/Shigella* was observed, while in FMT treatment the genus *Phascolarctobacterium* recovered to control levels after FMT administration (Fig. 2A,B).

### Virus shedding

**Figure 3:**
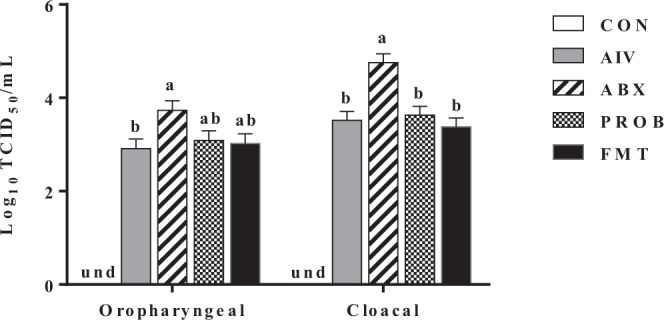
Avian influenza virus subytpe H9N2 shedding. Viral shedding was measured by tissue culture infectious dose 50/ml (TCID_50_/ml) in MDCK cells. Chickens were infected with 400 μl of 10^7^ TCID_50_/ml at 17 day of age through the oral-nasal route. Oropharyngeal and cloacal swabs (n = 9) were collected at day 5 post-infection. Treatments with different letters within a sample site differ significantly from each other (P < 0.05). Virus shedding from control group was undetected. und = undetected virus shedding.

At 5 days post-infection (p.i.), there was a higher H9N2 virus shedding in the oropharyngeal swabs of the ABX group compared to the group that was not administered with antibiotics but infected with H9N2 (AIV group) (P = 0.026, ANOVA), while PROB and FMT did not differ significantly compared to either AIV or ABX groups (P > 0.05, ANOVA). Furthermore, there was a significantly higher H9N2 cloacal virus shedding in the ABX group compared to all other treatments (P < 0.01, ANOVA), while both PROB and FMT groups did not show significant cloacal virus shedding compared to AIV group (P > 0.05, ANOVA) (Fig. 3).

### Type I interferons and IL-22 mRNA expression

**Figure 4:**
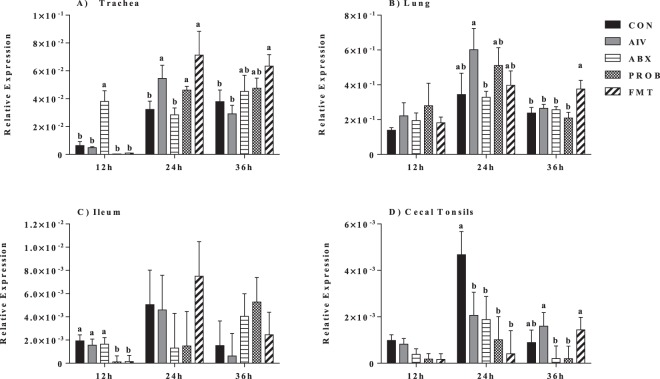
Relative expression of IFN-α in the trachea, lung, ileum and cecal tonsils. Samples (n = 6) were collected at 12 h, 24 h and 36 h post infection, and mRNA expression relative to β-actin was measured using quantitative real-time PCR. Bars with different letters at a time point differ significantly from each other (P < 0.05).

**Figure 5:**
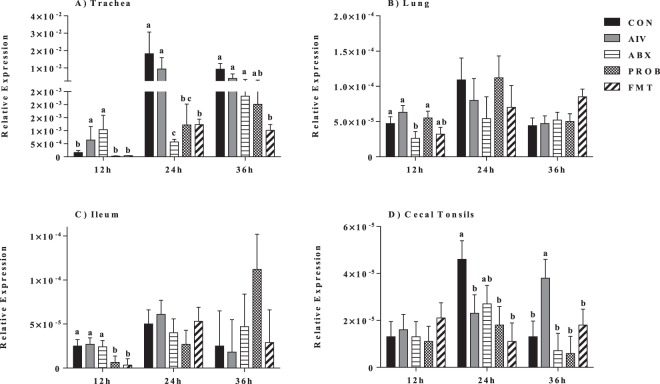
Relative expression of IFN-β in the trachea, lung, ileum and cecal tonsils. Samples (n = 6) were collected at 12 h, 24 h and 36 h post infection, and mRNA expression relative to β-actin was measured using quantitative real-time PCR. Bars with different letters at a time point differ significantly from each other (P < 0.05).

**Figure 6:**
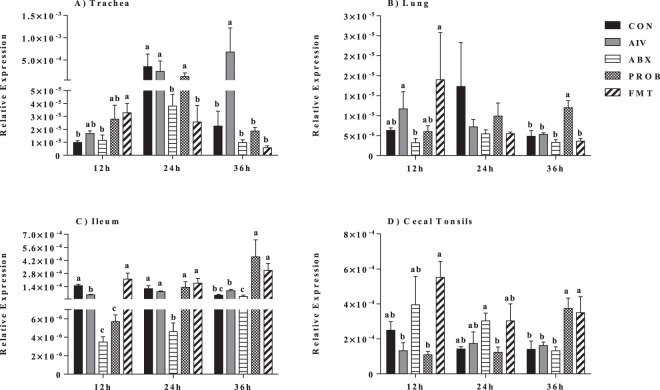
Relative expression of IL-22 in the trachea, lung, ileum and cecal tonsils. Samples (n = 6) were collected at 12 h, 24 h and 36 h post infection, and mRNA expression relative to β-actin was measured using quantitative real-time PCR. Bars with different letters at a time point differ significantly from each other (P < 0.05).

Expression of IFN-α in the respiratory tract (trachea and lung) showed that the administration of antibiotics resulted in the downregulation of this interferon compared to AIV group, which was mainly observed at 24 h p.i (P = 0.022, ANOVA). Furthermore, the administration of both PROB and FMT seemed to restore the expression of IFN-α, mainly at 24 h p.i., while other time points did not show significant differences (P > 0.05, ANOVA) (Fig. 4A,B). In the GIT (ileum and cecal tonsils), expression of IFN-α doesn’t seem to be altered at all time points, except in the cecal tonsils at 36 h p.i., where only FMT treatment restored the downregulated response observed in ABX treatment compared to AIV (P < 0.05, ANOVA) (Fig. 4C,D). Similarly, ABX treatment resulted in the downregulation of IFN-β expression in the trachea at 24 h p.i. and in the lung at 12 h p.i. (P < 0.05, ANOVA), while administration PROB and FMT resulted in restoration of the response to levels of the AIV treatment only in the lung at 12 h p.i. (Fig. 5A,B). Expression of IL-22 in the trachea (at 24 h and 36 h p.i.) and the lung (at 12 h p.i.) was significantly downregulated in the ABX treatment compared to AIV (P < 0.05, ANOVA), while both PROB and FMT treatments restored the expression levels to that of the AIV treatment, even though at different time points (Fig. 6A,B). Expression of IL-22 was consistently suppressed in the ileum in the ABX treatment when compared to AIV, while both PROB and FMT restored the expression to the level of AIV treatment, and at 36 h p.i. in PROB and FMT, higher IL-22 was observed compared to AIV (P < 0.05, ANOVA) (Fig. 6C). In the cecal tonsil, expression of IL-22 was inconclusive as ABX treatment did not results in significant difference compared to AIV, and both PROB and FMT showed higher expression of IL-22 compared to CON, AIV and ABX at 36 h p.i. (Fig. 6D).

### Histological Analysis

**Figure 7:**
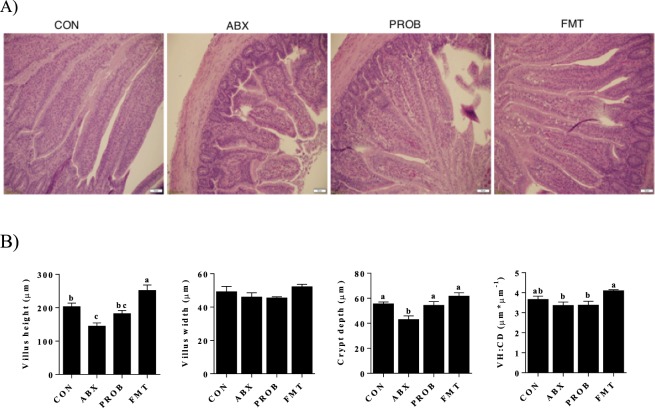
Morphometric analysis of ileum samples presented from day 16 of age (1 day before infection). (**A**) Hematoxylin and eosin (H&E) of the ileum samples. (**B**) Villus height, villus width, crypt depth, and villus height:crypt depth ration (VH:CD). Bars with different letters differ significantly from each other (P < 0.05).

Treatment of chickens with antibiotics resulted in a damaged general architecture of the ileum compared to control group, which was alleviated after the inoculation of probiotics and FMT (Fig. 7A). Furthermore, a significantly lower villus height was observed in chickens treated with antibiotics compared to both control and FMT treatments (P < 0.028, ANOVA), while no significant difference in villus width was observed among treatments (P > 0.05, ANOVA). A significantly lower crypt depth was also observed in the antibiotic treated chickens compared to all treatments (P < 0.031, ANOVA), while a significantly lower villus height:crypt depth ratio (VH:CD) was observed in both antibiotic treated chickens and chickens treated with probiotics after antibiotics compared to chickens treated with FMT (P < 0.025, ANOVA) (Fig. 7B).

### Correlations between cecal bacterial genera and cytokine responses

**Figure 8:**
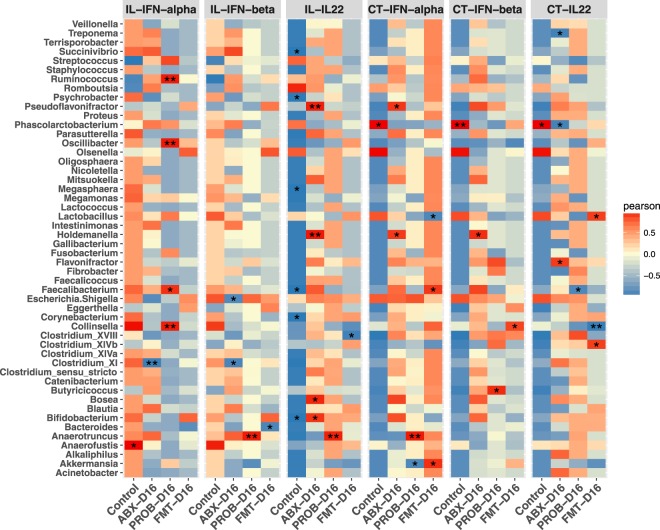
Correlogram showing Pearson’s correlations between bacterial genera and cytokine responses in the ileum and cecal tonsils. Pearson correlations were computed for the top 50 of bacterial taxa and average of relative expression of a cytokine measured in the ileum and cecal tonsils at 12 h, 24 h, and 36 h post infection. P-values were adjusted for false discovery rate according to Bonferroni and Hochberg procedure. An asterisk indicates a significance correlation between the bacterial taxa and the cytokine in a tissue (**P < 0.01, *P < 0.05).

Pearson correlation results are presented in Fig. 8. In the ileum, IFN-α was significantly and negatively correlated with *Clostridium* cluster XI in ABX group while in PROB group, it was significantly and positively correlated with *Collinsella*, *Faecalibacterium*, *Oscillibacter*, and *Ruminococcus*. Expression of IFN-β was significantly and negatively correlated with *Clostridium* cluster XI and *Escherichia/Shigella* in ABX group, while it was significantly and positively correlated with *Anaerotruncus* in PROB group, and significantly and negatively correlated with *Bacteroides* in FMT group. Expression of IL-22 was significantly and positively correlated with *Bifidobacterium*, *Bosea*, *Holdemanella* and *Pseudoflavonifractor* in ABX group, while in PROB group, it was significantly and positively correlated with *Anaerotruncus*, and significantly and negatively correlated with *Clostridium* cluster XVIII in FMT group.

In cecal tonsils, expression of IFN-α was significantly and positively correlated with *Pseudoflavonifractor* and *Holdemanella* in ABX group, while in PROB group it was significantly and negatively correlated with *Akkermansia* and significantly and positively correlated with *Anaerotruncus*. In FMT group, expression of IFN-α was significantly and negatively correlated with *Lactobacillus* while it was significantly and positively correlated with *Akkermansia* and *Faecalibacterium*. Expression of IFN-β was significantly and positively correlated with *Holdemanella* in ABX group. Furthermore, expression of IFN-β was significantly and positively correlated with *Butyricoccus* and *Collinsella* in PROB and FMT groups, respectively. Expression of IL-22 was significantly and negatively correlated with *Phascolarctobacterium* and *Treponema* in ABX group, and significantly and positively correlated with *Flavonifactor*. In PROB group, IL-22 expression was significantly and negatively correlated with *Faecalibacterium* while it was significantly and negatively correlated with *Collinsella* and significantly and positively correlated with *Lactobacillus* and *Clostridium_XIVb* in FMT group.

## Discussion

Avian influenza virus subtype H9N2 has tropism for several tissues, including tissues of the upper respiratory tract and GIT of chickens^8^. This virus subtype has received recent attention as it has previously reached panzootic proportions, and despite continuous vaccination of chickens, H9N2 vaccines have become less effective^23,24^. Furthermore, this virus has provided internal genes to novel HPAI virus strains with potential significant impact on human health^24^. Since most viral infections occur via the mucosal route and encounter commensal microbiota inhabiting the mucosal surfaces, recent studies have suggested that commensal gut microbiota plays an important role in viral pathogenesis. The current study was conducted to investigate the role of commensal gut microbiota of chickens in innate immunity against avian influenza virus infection.

In the current study, next-generation sequencing using the Illumina MiSeq® platform and analysis showed that using an antibiotic cocktail to induce dysbiosis shifted the composition while administration of probiotic bacteria and FMT was not able to restore it to its original composition. In mice, similar findings have been observed where, even though the diversity improves after FMT, a complete recovery of the gut microbiota compared to that of untreated mice was not observed^14^. In contrast, other studies reported that the administration of FMT after antibiotic depletion resulted in the reversion of the gut microbiota to its original composition in rhesus macaques (*Macaca mulatta*)^25^. Differences in these findings may be associated with the methodologies used and the species under study. Furthermore, higher diversity was observed in antibiotics treated chickens in the current study. While diversity and richness typically decrease with antibiotics, the disruption of the microbiota may have enabled environmental bacteria to colonize resulting in increased richness and diversity.

Virus shedding results from the current study, where the depletion of gut microbiota resulted in higher virus shedding compared to chickens with intact microbiota, are in agreement with our previous work, where chickens treated with antibiotics to deplete their gut microbiota shed significantly more virus compared to undepleted chickens^19^. When gut microbiota depleted chickens were treated with a cocktail of probiotics or FMT, both oropharyngeal and cloacal shedding were reversed to levels not significantly different from those of the undepleted group, suggesting that the reconstitution of gut microbiota of chickens, via different mechanisms, resulted in restored capacity of the host to resist virus infection.

Type I IFNs, which include IFN-α and IFN-β, play an important role in innate responses to viral infections by inducing an anti-viral state in virus-infected and uninfected cells as well as in bystander cells, interfering in the various stages of viral life cycle by mechanisms involving degradation of viral nucleic acids or inhibition of viral gene expression^26–28^. In the current study, the expression of IFN-α and IFN-β in the GIT and respiratory tract was down-regulated in antibiotic depleted chickens mainly at 24 h and 36 h p.i., respectively. Previously, we showed that depletion of the gut microbiota resulted in compromised type I IFN response to AIV subtype H9N2 infection of chickens in a similar manner^19^. A study in mice showed that antibiotic depletion resulted in impaired expression of type I IFNs and their inducible associated antiviral response genes, such as IFN-β, interferon regulatory factor 7 (IRF7), interferon-inducible nuclear protein MX1, and 2′5′-oligoadenylate sythetase (OAS), against influenza virus and cytomegalovirus resulting in inability of clearance of the viruses^29,30^. When chickens were treated with either the lactobacilli probiotics or the FMT following antibiotic treatment, type I IFN responses were restored to the levels observed in chickens that received no antibiotics, even though the levels of type I IFNs recovery were not consistently observed in the time points assessed suggesting that complete restoration of this mechanism may not be realized with the models of microbial restoration used in the current study.

Among the major beneficial roles of commensal gut microbiota are maintenance of a normal homeostasis by modulating host immunity, development and physiology. A cytokine that is closely associated with cellular proliferation, tissue protection and regeneration, and inflammation is IL-22, which is a member of the IL-10 family of cytokines^31^. IL-22R, which is constitutively expressed by various epithelial cells, and its ligand, IL-22, are important in the maintenance and homeostasis of GIT epithelial cells^32^. IL-22 along with IFNs can synergistically control viral infections in the GIT in a IFN receptor signaling and STAT1 dependent manner^33^. In the current study, significant downregulation in the expression of IL-22 was observed in all tissues of antibiotic depleted chickens, except at 36 h p.i. in the cecal tonsils. Especially in the ileum, the expression of this gene was highly suppressed in the depleted chickens. However, upon treatment with probiotics or FMT, the recovery of IL-22 expression was significant, and in both the ileum and CT, this recovery was to a level similar to that of undepleted chickens. Therefore, our findings suggest that gut microbiota of chickens is highly involved in modulating the expression of IL-22 in both the upper respiratory system and the GIT. More importantly, after H9N2 infection, the expression of IL-22 in the probiotic or FMT groups was significantly higher compared to undepleted chickens. This raises the possibility that intact gut microbiota can induce the expression of IL-22 after AIV infection in chickens. It has been reported that inoculation of cecal contents from 3-weeks-old chickens to day-old chickens followed by *Salmonella* Enteritidis infection was associated with a significantly higher IL-22 expression and the reduction of *S*. Enteritidis counts in the cecum^34^. Another study by Volf *et al*.^35^ reported that raising germ-free chickens under conventional conditions or inoculating them with a tetraflora containing *E*. *coli*, *Enterococcus faecium*, *Lactobacillus rhamnosus* and *Clostridium butyricum* induced the expression of IL-22. Histomorphological analysis in the current study also showed strong recovery in the general architecture of the ileum after probiotic or FMT administration. Coordinated responses between the gut microbiota and IL-22 in the GIT are known to regulate and maintain the GIT epithelial barrier functions^36^. In germ-free mice, IL-22 response was severely impaired in cells isolated from the small intestine, and mice were more susceptible to a bacterial infection with *Citrobacter rodentium*^37^. Our findings suggest that the gut microbiota of chickens may enhance innate immunity to avian influenza virus subtype H9N2 in mechanisms that involves tissue remodeling and regeneration and the expression of key cytokines such as IL-22.

In chickens treated with antibiotics, there was a negative correlation of IFN-α expression with the presence of *Clostridium* cluster XI in the ileum. *Clostridium* cluster XI is a major source of the metabolite deoxycholic acid (DCA) that has previously been shown to be reduced in mice treated with a cocktail of antibiotics similar to that used in the current study^38^. DCA and other bile acid metabolites, which can be regulated by interferons, are known to enhance viral infection by inhibiting OAS activity, which is critical for the induction of degradation of viral and cellular RNAs thereby blocking viral infections^39,40^. Even though there was no positive or negative correlation with lactobacilli in the probiotic group, there was a strong positive correlation between IFN-α, and *Collinsella* and *Faecalibacterium*. A member of the *Coriobacteriaceae* family, *Collinsella* has been shown to be strongly and positively correlated with IFN responses and protection against rotavirus infection in the GIT of pigs^41,42^. Previously, *L*. *acidophilus*, *L*. *reuteri*, and *L*. *salivarius* from the same source used in the current experiment resulted in differential cytokine expressions in the cecal tonsils of chickens with *L*. *salivarius* inducing a more anti-inflammatory response^43^. In humans, infection with H7N9 virus was associated with significant reduction of *Faecalibacterium*^44^. Therefore, in the current study, an increase in this genus in correlation with the upregulation of IFN-α may be associated with the virus reduction in chickens treated with probiotics. A consistent strong positive correlation was observed between the expression of IFN-α, IFN-β, and IL-22 in the GIT and the genus *Anaerotruncus* in chickens treated with probiotics after antibiotic depletion. Previously, this genus has been associated with the expression of acetyl-CoA acetyltransferase, 3-hydroxyacyl-CoA dehydrogenase and enoyl-CoA hydratase in chicken GIT, which are enzymes necessary for butyrate production^45^. In chickens treated with FMT, a positive correlation was observed between the expression of IL-22 in the ileum and cecal tonsil and the genera *Lactobacillus* and *Clostridium* cluster *XIVb*. In mice, IL-22 deficiency was associated with decreased abundance of genus *Lactobacillus*, while higher abundance of the genus *Lactobacillus* was associated with restoration of the epithelial barrier integrity and higher IL-22 production by gut resident innate lymphoid cells in mice^46,47^. *Clostridium* cluster *XIVb* are butyrate producing bacteria and accounted for almost 60% of the mucin-adherent microbiota in a previous study^48,49^, and this might be associated with the recovery of the ileal morphology observed in the current study, even though more studies are required to establish this observation.

In conclusion, our findings suggest that a shift in the composition of commensal gut microbiota of chickens using antibiotic treatment results in a compromised innate response with different mechanisms being involved including type I IFNs, IL-22 and tissue barrier function. Reconstitution of the gut microbiota of chickens with probiotic combinations or FMT resulted in the recovery of the different innate response mechanisms following H9N2 infection. Therefore, gut microbiota composition of chickens may play an important role in immunity of chickens against influenza viruses. Future research looking at the role of probiotics or other intervention mechanisms that modulate gut microbial composition of undepleted chickens is warranted to observe whether modulation of the gut microbiota can be used in minimizing influenza virus outbreaks in poultry.

## Materials and Methods

### Experimental design

All experimental procedures were approved by the University of Guelph Animal Care Committee and all methods were carried out in accordance with the Canadian Council on Animal Care Guidelines. A total of 100 one-day-old specific pathogen free (SPF) layer chickens (CFIA, Ottawa Laboratory, Nepean, ON, Canada) were randomly assigned to five treatment groups with 20 chickens per treatment. Chickens were kept in Horsfall isolation units in a Biosafety level II isolation facility at the University of Guelph, which provided caging of individual treatments to avoid cross contamination. Antibiotic-free feed was provided *ad libitum*. The treatment groups included a control group with chickens that were neither treated with cocktail of antibiotics nor infected with H9N2 virus (CON), a group that was not treated with antibiotics but was infected with H9N2 virus (AIV), a group that was treated with a cocktail of antibiotics and infected with H9N2 virus (ABX), a group that was treated with antibiotics and was administered probiotics combination and infected with H9N2 virus (PROB), and a group that was treated with antibiotics, treated with FMT suspension and infected with H9N2 virus (FMT). Five chickens per group were used for microbial composition analysis, 6 chickens per group were used for gene-expression analysis and 9 chickens per treatment were used for virus shedding.

### Antibiotics, probiotics and fecal microbial transplant administration

Chickens were gavaged twice daily, starting at day 1 of age, with a cocktail of antibiotics in 10 mL of water per kg of body weight. The cocktail of antibiotics contained 5 mg/ml vancomycin, 10 mg/ml neomycin, 10 mg/ml metronidazole and 0.1 mg/ml amphotericin-B while 1 g/l of ampicillin was continuously provided in drinking water. Chickens in ABX treatment received antibiotics for 16 days, while chickens in PROB and FMT treatments were treated with antibiotics for 12 days followed by administration of either probiotic combination or FMT for 4 days starting 12 hrs after termination of antibiotic treatment. Chickens in PROB group were administered with a cocktail of five bacterial isolates from intestines of healthy chickens, which included *Lactobacillus salivarius*, *L*. *johnsonii*, *L*. *reuteri*, *L*. *crispatus*, and *L*. *gasseri*. Each isolate was first individually grown in De Man, Rogosa and Sharpe (MRS) medium overnight and 10^9^ CFU/ml of each were mixed, and 1 mL of this cocktail was administered daily for four days to each chicken by gavaging to the crop using a 1 mL syringe. Preparation and administration for FMT was conducted according to Li *et al*.^50^ where 8 gm of cecal contents from age-matched chickens was homogenized in 10 ml of sterile PBS and centrifuged at 1600 × g for 30 seconds at 4 °C to pellet the particulate matter. Donor chickens were of same origin and raised in floor pens and fed antibiotic free diets. Supernatant was collected and optical density (OD) measured. An OD of 0.5 represented 10^8^ cells and each chicken was gavaged with 1 × 10^9^ bacterial cells every day for four days post antibiotic treatment.

### DNA extraction, 16S rRNA gene sequencing and tissue processing

Cecal contents were collected at day 16 of age prior to infection of chickens and was immediately frozen at -80 °C. To compare differences between day 12 (ABX-D12) and 16 (ABX-D16) of age, samples were collected from 5 chickens from each group to assess if chickens in ABX-D12 recovered their microbiota by day 16 of age. Furthermore, cecal contents were collected from 5 chickens per group to compare microbial composition between control, ABX-D16, PROB (PROB-D16), and FMT (FMT-D16). Microbial genomic DNA extraction was performed using QIAamp DNA Stool mini kit (Qiagen, Toronto, Canada) according to the manufacturer’s instructions. DNA concentrations were measured with a Qubit while DNA quality was assessed with a NanoDrop^®^ ND-1000 spectrophotometer (NanoDrop Technologies, Wilmington, DE). The V4 hypervariable region of the 16S rRNA gene was PCR-amplified and sequenced on Illumina MiSeq (Illumina, San Diego, CA) using a dual-indexing strategy for multiplexed sequencing developed at the University of Guelph’s Genomics Facility, Advanced Analysis Centre (Guelph, Ontario, Canada) as described previously^51^. Sequences were curated using Mothur v.1.36.1 as described in the MiSeq SOP^52^. Sequence data processing and analysis were performed as described previously^9^. Briefly, after generation of contigs and merging of duplicate sequences, alignment of non-redundant sequences to customized references of the SILVA 102 bacterial database^53^ was performed using the align.seqs command. Non-redundant aligned reads were created using unique.seqs command followed by the removal of chimeric sequences using the chimera.uchime^54^ and remove.seqs commands. Clustering of sequences into OTUs was then performed using the dist.seqs and cluster commands, followed by conversion to.*shared* format using the make.shared command. Taxonomy was also assigned to each sequence using the Ribosomal Database Project (RDP) bacterial taxonomy classifier. All OTU-based analyses were performed in Mothur. Alpha- and beta-diversity analyses were performed in Mothur, and non-metric multi-dimensional scaling (NMDS) was performed with Bray Curtis dissimilarities with analysis of molecular variance (AMOVA) performed to assess the significant difference in spatial separation among treatments. Identification and visualization of taxa with differential abundance was performed using the linear discriminant analysis (LDA) effect size (LEfSe) method for high-dimensional class comparisons. Treatment groups were assigned as comparison classes and LEfSe identified features that were statistically different between treatments were then compared using the non-parametric factorial Kruskal-Wallis (KW) sum-rank test and Linear Discriminant Analysis (LDA) >4^55^. Tissue sections of the ileum at day 16 of age from control, ABX, PROB and FMT were fixed in 10% neutral-buffered formalin, embedded in paraffin, sectioned at a thickness of 5 μm, and stained with hematoxylin and eosin (H&E) at the Animal Health Laboratory (AHL), University of Guelph, Ontario.

### Virus propagation and infection of chickens

Embryonated SPF eggs (CFIA, Ottawa Laboratory, Nepean, ON, Canada) were incubated at 37 °C for 10 days followed by inoculation with 4 hemagglutination (HA) units/egg of virus, A/turkey/Wisconsin/1/1966(H9N2), and further incubated for 72 hours at 35 °C. Eggs were then refrigerated at 4 °C overnight followed by collection of allantoic fluid, which was then centrifuged at 400 × g for 5 minutes and stored at -80 °C until further use. Chickens in the AIV, ABX, PROB and FMT groups were challenged at 17 days of age via the oral-nasal route with 400 μl of 10^7^ tissue culture infectious dose 50 (TCID_50_/mL). Cloacal and oropharyngeal swabs were collected from 9 chickens per group at day 5 post infection (p.i.), and TCID_50_ was used to determine virus shedding, where a serial (1:2 dilution) of samples were used to infect Madin-Darby Canine Kidney (MDCK) cells. Day 5 p.i. was selected for sampling of oropharyngeal and cloacal swabs since we found this to be the peak of virus shedding in a previous study^19^.

### RNA extraction, cDNA synthesis and quantitative real-time PCR

Six chickens per treatment were euthanized at 12, 24, and 36 hours p.i., and tissues from the trachea, lung, ileum and cecal tonsils were collected in RNAlater and stored at -80 °C until extraction of RNA. Tissue samples (50–100 mg) were homogenized in Trizol and RNA was extracted according to the manufacturer’s instructions (Trizol®, Life Technologies, Inc.). RNA quantity and quality were determined using the NanoDrop^®^ ND-1000 spectrophotometer (NanoDrop Technologies, Wilmington, DE). cDNA synthesis was carried out by reverse transcription of 500 ng of total RNA using Oligo(dT)12–18 primers and the Super-Script^TM^ First-Strand Synthesis System (Life Technologies, Inc.) according to manufacturer’s instructions. Quantitative real-time polymerase chain reaction (qRT-PCR) was run in 384-well plates with 5 μL of cDNA (1:10 dilution), 0.25 μM of forward and reverse primers, and 10 μL of SYBR Green (Roche Diagnostic, Laval, QC, Canada) and a balance of water to 20 μL total reaction volume per well. Primer sequences and their respective annealing temperatures are provided in Table 1.

**Table 1:** Primer sequences used in qRT-PCR runs.

Gene	Sequences	Annealing temp. (°C)	Reference
β-actin	F: CAACACAGTGCTGTCTGGTGGTA	60	^57^
	R: ATCGTACTCCTGCTTGCTGATCC		
IFN-α	F: ATCCTGCTGCTCACGCTCCTTCT	64	^57^
	R: GGTGTTGCTGGTGTCCAGGATG		
IFN-β	F: GCCTCCAGCTCCTTCAGAATACG	64	^58^
	R: CTGGATCTGGTTGAGGAGGCTGT		
IL-22	F: TCAACTTCCAGCAGCCCTACAT	60	^59^
	R: TGATCTGAGAGCCTGGCCATT		

### Statistical analysis

All data for qRT-PCR, where relative expression of each gene was calculated relative to β-actin as a house-keeping gene, were analyzed using the Mixed procedure of SAS^®^ (SAS Institute, Inc., Cary, NC) with animal as an experimental unit. Post-hoc ANOVA was performed, and means were considered significantly different at a P < 0.05, and summary of P-values for multiple comparisons are presented as P < 0.01 and P < 0.05. When data was not normally distributed it was log-transformed. Pearson correlation method was used to assess the potential link between significant changes in genus level microbial composition in the ceca induced by treatments and cytokine responses in both the ileum and cecal tonsils post H9N2 infection, and P-values were adjusted for false discovery rate using the Benjamini and Hochberg procedure^56^.

## Data Availability

The datasets generated and analyzed during the current study are available from the corresponding author on reasonable request.
