# Author Correction: Gut microbiota-mediated protection against influenza virus subtype H9N2 in chickens is associated with modulation of the innate responses

**DOI:** 10.1038/s41598-018-34065-8

**Published:** 2018-10-31

**Authors:** Alexander Yitbarek, Khaled Taha-Abdelaziz, Douglas C. Hodgins, Leah Read, Éva Nagy, J. Scott Weese, Jeff L. Caswell, John Parkinson, Shayan Sharif

**Affiliations:** 1 Department of Pathobiology, Ontario Veterinary College, University of Guelph, Ontario, N1G 2W Canada; 2 Pathology Department, Faculty of Veterinary Medicine, Beni-Suef University, Al Shamlah, 62511 Beni-Suef, Egypt; 3 Department of Computer Science, University of Toronto, Toronto, M5S 3G4 Canada; 4 Division of Molecular Structure and Function, Research Institute, Hospital for Sick Children, Toronto, Ontario M5G 1X8 Canada; 5 Departments of Biochemistry and Molecular Genetics, University of Toronto, M5S 1A8 Toronto, Canada

Correction to: *Scientific Reports* 10.1038/s41598-018-31613-0, published online 04 September 2018

The original version of this Article omitted an affiliation for Khaled Taha-Abdelaziz. The correct affiliations for Khaled Taha-Abdelaziz are listed below:

Department of Pathobiology, Ontario Veterinary College, University of Guelph, Guelph, ON N1G 2W1, Canada.

Pathology Department, Faculty of Veterinary Medicine, Beni-Suef University, Al Shamlah, Beni-Suef, 62511, Egypt.

This has now been corrected in the HTML and PDF versions of this Article.

